# Are viral vector-mediated therapies compatible with aberrant glycosylation?

**DOI:** 10.1016/j.omtm.2025.101540

**Published:** 2025-07-22

**Authors:** I.J.J. Muffels, R. Budhraja, S. Radenkovic, R. Shah, A. Pandey, E. Morava, T. Kozicz

**Affiliations:** 1 Department of Genetic and Genomic Sciences, Icahn School of Medicine at Mount Sinai, 1425 Madison avenue, New York, NY 10029, USA; 2 Department of Laboratory Medicine and Pathology, Mayo Clinic, 200 1st St SW, Rochester, MN 55905, USA; 3 Zydus Research Center, Zydus Lifesciences Limited, S.G. Highway 536, Ahmedabad, Gujarat 382481, India; 4 Department of Metabolic Diagnostics, University Medical Center Utrecht, Heidelberglaan 100, 3584 CX, Utrecht, the Netherlands; 5 Center for Individualized Medicine, Mayo Clinic, 200 1st St SW, Rochester, MN 55905, USA; 6 Department of Biophysics, University of Pecs Medical School, Szigeti út 12, 7624 Pécs, Hungary; 7 Department of Anatomy, University of Pecs Medical School, Szigeti út 12, 7624 Pécs, Hungary

**Keywords:** congenital disorders of glycosylation, CDGs, adeno-associated virus, viral vector, glycosylation, glycoproteomics, neurodegenerative diseases

## Abstract

The ability of adeno-associated viruses (AAVs) to transduce host cells relies on interactions with glycan moieties on the cellular surface. Consequently, disrupted protein glycosylation, which is seen in a range of neurodevelopmental and neurodegenerative diseases, could impair transduction efficiency. Understanding how altered glycosylation impacts AAV binding is essential to optimize AAV-mediated therapeutic strategies. We used glycoproteomics data from cortical brain organoids and iCardiomyocytes of individuals with congenital disorders of glycosylation (CDG) (ALG13-, PMM2-, and PGM1-CDG) to examine the abundance of AAV-binding glycan species. Additionally, we assessed the abundance of coreceptors in proteomics data. We found that the abundance of AAV-binding glycan species was downregulated for all CDG subtypes, but this was significant only for AAV5-, AAV8-, and AAV9-binding glycan motifs in PGM1-CDG. The proteomics data showed significantly decreased abundance of the coreceptor PDGFRβ in ALG13-CDG. The downregulation of glycan species and AAV coreceptors in models of aberrant protein glycosylation underscores the need to optimize AAV selection for conditions with altered protein glycosylation, including CDG and neurodegenerative diseases such as Parkinson’s and Alzheimer’s disease.

## Graphical abstract

**Figure:**
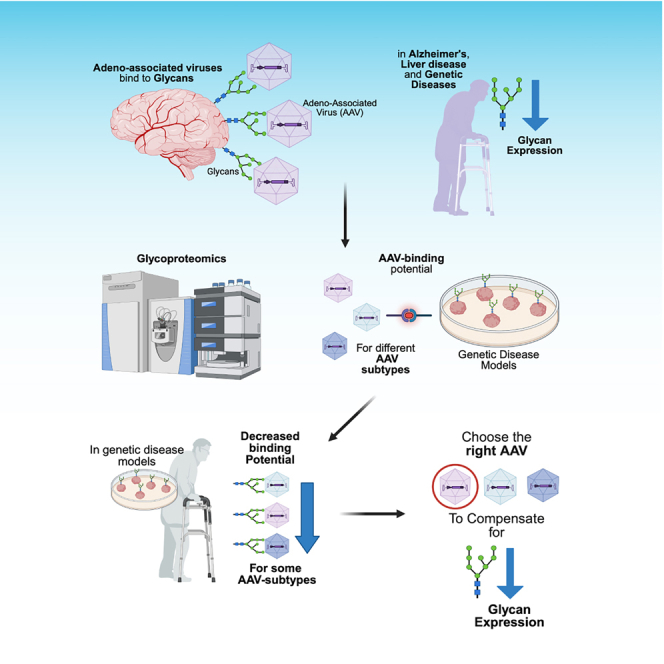

## Teaser

Muffels and colleagues demonstrate here that altered glycosylation, which is observed in congenital-, liver-, and neurodegenerative disorders, can reduce AAV-binding glycan expression, potentially impairing transduction efficacy. These findings underscore the need for glycosylation-aware vector selection in AAV-therapies for disorders with glycosylation defects, such as CDGs and Alzheimer’s disease.

## Introduction

Adeno-associated viruses (AAVs) are versatile, non-pathogenic viral vectors, which are widely used for delivering genetic material to specific tissues. Advancements in AAV design and delivery have enabled their success in treating rare genetic conditions, such as spinal muscular atrophy, hemophilia, and retinal dystrophies, and as genetic vaccines targeting diseases such as Alzheimer’s disease.^1^^,^^2^^,^^3^^,^^4^^,^^5^

AAVs rely on specific glycan species for cellular entry, along with sufficient expression and glycosylation of coreceptors for successful transduction.^6^ However, many diseases that have been trialed for AAV-based therapies, including Alzheimer’s disease,^7^ Parkinson’s disease,^8^ chronic liver diseases,^9^ and congenital disorders of glycosylation (CDG),^10^ are associated with aberrant glycosylation. These glycosylation defects may impair AAV binding and transduction efficiency, yet this critical issue remains largely unexplored.

In this study, we utilized glycoproteomic data from cortical brain organoids and iCardiomyocytes from individuals with CDG as a model of aberrant glycosylation, to investigate how altered glycan composition and coreceptor abundance impact AAV uptake. Our analysis revealed significant changes in glycan species essential for AAV binding, along with variations in coreceptor abundance. These findings highlight the need for tailored AAV vector selection to optimize gene therapy outcomes, not only for rare glycosylation disorders but also for prevalent conditions with secondary glycosylation defects, such as Alzheimer’s disease.

## Results

For this study, we included glycoproteomics data of patients with PMM2-CDG, the most common CDG, with neurological symptoms consistently present in all cases.^11^ We also included data from ALG13-CDG and PGM1-CDG, two other common CDG types, which manifest in one specific tissue type, i.e., the brain (ALG13-CDG) and heart/muscle (PGM1-CDG). PMM2-CDG results in underglycosylated proteins, which is reflected in a type I CDG serum transferrin profile.^12^ Intriguingly, even though ALG13-CDG is considered a type I CDG, it does not present with altered serum transferrin profiles in most patients.^13^^,^^14^ PGM1-CDG results in a mixed pattern of both type I CDG and type II CDG defects due to altered uridine diphosphate (UDP)-galactose metabolism, which hampers glycan processing in the Golgi system.^15^ The underlying genetic defects and age of the patients are detailed in Table 1.

**Table 1:** Showing the genetic variants, age when biopsies were obtained, transferrin isoelectric focusing and gender of the patients included in this study

Donor	Genetic defect	Age/sex	Transferrin isoelectric focusing	AAV1/6	AAV5	AAV9
I1	*PMM2* c.422G>A; c.647A>T. p.R141H, p.N216I	9 yo/M	mono-oligo/di-oligo ratio 0.31 (<0.06) A-oligo/di-oligo ratio 0.006 (<0.011) tri-sialo/di-oligo ratio 0.04 (<0.05)	0.655	0.585	0.629
I2	*PMM2* c.422G>A; c.415G>A, p.R141h, p.E139K	9 yo/F	mono-oligo/di-oligo ratio 0.07 (<0.06) A-oligo/di-oligo ratio 0.040 (<0.011) tri-sialo/di-oligo ratio 0.03 (<0.05)	0.968	0.841	0.887
I3	*PMM2* c.422G>A; c.548T>C, p.P183S, p.R141H	8 yo/M	mono-oligo/di-oligo ratio 0.31 (<0.06) A-oligo/di-oligo ratio 0.029 (<0.011) tri-sialo/di-oligo ratio 0.05 (<0.05)	0.939	0.757	1.06
11816	*ALG13* c.320A>G, p.N107S	4 yo/M	all within normal range	0.763	0.28	0.829
11740	*ALG13* c.320A>G, p.N107S	7 yo/F	all within normal range	1.04	0.315	0.992
12106	*ALG13* c.320A>G, p.N107S	9 yo/F	all within normal range	1.05	0.364	1.02
P1	*PGM1* c.551delT homozygous, p.F184Sfs∗9	3 yo/F	mixed type I/II pattern	0.876	1.04	1.07
P2	*PGM1* c.1162 G>A; c.1547 T>C, p.Glu388Lys, p.Leu516Pro	10 yo/M	mixed type I/II pattern	0.733	1.11	0.868
P3	*PGM1* c.1508 G>A homozygous, p.Arg503Gln	16 yo/F	mono-oligo/di-oligo ratio 0.19 (<0.06) A-oligo/di-oligo ratio 0.009 (<0.011) tri-sialo/di-oligo ratio 0.11 (<0.05)	0.73	0.676	0.799

On the right, the average foldchange values of the extracellular glycan species binding to each specific AAV subtype are shown. yo, years old; IEF, transferrin isoelectric focusing.

### Assessing binding of extracellular glycan species to different AAV subtypes

We studied tissue tropism for each AAV serotype and the specific glycan species they bind to (Table 2).^16^ Next, we searched for N-glycan species in the glycoproteomics datasets of individuals with CDG that expressed the specific AAV-binding terminal glycan extracellularly. Since O-glycoproteomics data of disease-relevant tissues is not available for patients with CDG, we focused on N-glycosylation only. Similarly, heparate sulfate proteoglycans were not present in the glycoproteomics datasets and were not assessed. Consequently, our analysis focused on AAV1, AAV5, AAV6, AAV8, and AAV9 serotypes, which specifically bind to N-glycan species.

**Table 2:** Different AAV subtypes and the glycan species they bind to

AAV serotype	Tissue tropism	Primary binding site	Specific primary N-glycan receptor	Coreceptor
AAV1	skeletal muscle, heart, and CNS	N-linked sialic acid	A terminal α2-3-linked sialic acid linked to N-acetylgalactosamine and an additional β1-4-linked N-acetylglucosamine.	integrin αvβ5
AAV2	liver and kidney	heparan sulfate proteoglycans and heparate sulfate chains (N-acetylglucosamine and iduronic acid residues)	N/A	integrin αvβ5, FGFR1, hepatocyte growth factor receptor (HGFR), Laminin receptor, CD9
AAV3	lung, liver, heart, and lung	heparan sulfate proteoglycans and heparate sulfate chains (N-acetylglucosamine and iduronic acid residues)	N/A	FGFR1, laminin receptor.
AAV4	CNS, heart, and lung	O-linked sialic acid	N/A	N/A
AAV5	CNS and lung	N-linked sialic acid	A terminal α2-3-linked sialic acid, linked to a β1-4-linked galactose in the second position, followed by N-acetylglucosamine.	PDGF receptor (PDGFRβ)
AAV6	skeletal muscle, heart, liver, lung, and CNS	N-linked sialic acid	A terminal α2-3-linked sialic acid linked to an N-acetylgalactosamine and an additional β1-4-linked N-acetylglucosamine.	EGF receptor
AAV8	liver, heart, CNS, and skeletal muscle	N-linked terminal galactose	all N-linked glycans with terminal galactoses	laminin receptor
AAV9	liver, heart, CNS, and skeletal muscle	N-linked galactose	all N-linked glycans with terminal galactoses	laminin receptor

**Figure 1:**
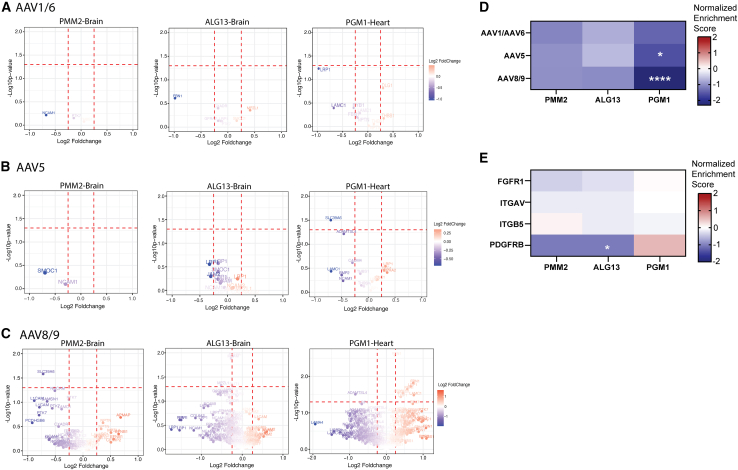
Volcano plot showing the abundance of AAV-binding glycan species and coreceptors for PMM2-CDG (3 patients and 2 healthy controls), ALG13-CDG (3 patients and 3 healthy controls), and PGM1-CDG (3 patients and 4 healthy controls) The red line on the *y* axis corresponds with *p* values below 0.05. The red lines on the *x* axis corresponds with log2 fold changes of >0.25 of < −0.25. (A) Volcano plot showing the abundance of AAV1/AAV6-binding glycan species. (B) Volcano plot showing the abundance of AAV5-binding glycan species. (C) Volcano plot showing the abundance of AAV8/AAV9-binding glycan species. (D) Heatmap summarizing the results from Figure 1C. The scale reflects the normalized enrichment, which was determined through GSEA. The stars reflect the adjusted *p* value (FDR). ∗*p* < 0.05, ∗∗*p* < 0.01, ∗∗∗*p* < 0.001, and ∗∗∗∗*p* < 0.0001. (E) Heatmap showing the abundance of AAV coreceptors in different CDG.

We identified a limited number of extracellular N-glycan motifs harboring the specific glycan conformation where AAV1 and AAV6 could bind to (Figures 1A and 1D). However, the majority of these glycoproteins were downregulated in PMM2-, ALG13-, and PGM1-CDG patient-derived cells (normalized enrichment score [NES] = −1.1, −0.75, and −1.4, respectively) although not significantly.

For AAV5, we found the majority of glycan species were downregulated; for PMM2, NES was −1 (*p* = 0.4), and for ALG13-CDG, NES was −0.63 (*p* = 0.9) (Figures 1B and 1D). The downregulation of AAV5-binding glycan species was significant for PGM1-CDG, where NES was −1.6 (*p* = 0.04) (Figures 1B and 1D).

To assess potential binding of AAV8/AAV9 in patient-derived cells with CDG, we assessed the abundance of non-sialyated glycan species. For ALG13-and PMM2-CDG, non-sialyated glycoproteins with terminal galactose were downregulated (NES = −1.01, *p* = 0.5 and NES = −1.05, *p* = 0.3, respectively) (Figures 1C and 1D). For PGM1-CDG, non-sialyated glycoproteins with terminal galactose residues were downregulated significantly (NES = −2.3, *p* < 0.0001) (Figures 1C and 1D). Thus, we found downregulation of AAV-binding glycan species for all the AAV subtypes assessed (AAV1, 5, 6, 8, and 9), but this was not significant for PMM2-and ALG13-CDG. For PGM1-CDG, we found that the abundance of both AAV5- and AAV8/9-binding glycan species were significantly decreased.

### Abundance of protein factors that mediate efficient AAV entry and transduction

Several transmembrane and vesicle trafficking proteins are essential for the efficient transduction of human cells.^6^ Interestingly, pathogenic variants in some of these 75 essential proteins, including EXT1, EXT2, PGAP2, and COG7, are associated with CDG.^17^ This led us to question whether other types of CDG, not necessarily involving variants in these specific genes, might exhibit altered abundance of these proteins. We found that in PMM2- and PGM1-CDG, the abundance of these proteins was significantly upregulated, potentially as a mechanism to compensate for faulty glycosylation (NES = 1.4, *p* = 0.03 and NES = 1.4, *p* = 0.04), whereas in ALG13-CDG, these proteins were not significantly altered (NES = −0.98, *p* = 0.9). We also assessed the abundance of AAV coreceptors in our proteomics data. We found that the PDGFRβ (AAV5) coreceptor showed significantly decreased abundance in ALG13-CDG (Figure 1E). Therefore, the transduction efficiency of these specific subtypes might be impacted. However, functional validation is needed to confirm whether the expression of these proteins is truly altered and to assess their potential role in influencing transduction efficiency. Additionally, it should be noted that coreceptor expression generally exerts only a limited effect on AAV-binding efficiency.

## Discussion

In this study, we utilized CDG as a model of aberrant protein glycosylation to examine how alterations in glycan composition and the abundance of protein coreceptors affect transduction efficiency. In PGM1-CDG, the abundance of AAV5-, AAV8-, and AAV9-binding glycan species was significantly reduced, while in other CDG types, the reductions were present but not statistically significant. Additionally, the abundance of AAV coreceptor PDGFRβ was significantly reduced in ALG13-CDG. These results suggest that aberrant glycosylation could impair AAV5-, AAV8-, and AAV9 binding, while reduced coreceptor abundance could mildly affect the cellular entry of AAV5 in ALG13-CDG.

The CDG included in this study mostly affect the first steps of glycosylation in the Endoplasmic Reticulum (ER), although PGM1-CDG also affects UDP-galactose import into the Golgi system, thereby explaining the downregulation of glycan species with terminal galactoses specific to PGM1-CDG.^18^ However, the inclusion of a CDG that solely affects late-stage Golgi system glycosylation could shed on the entire spectrum of glycosylation defects, as these defects usually lead to a decrease in glycan species with terminal sialic acids, and an increase in terminal galactoses. Similarly, in secondary glycosylation defects, such as chronic liver disease, glycan sialylation is predominantly affected, which may reduce the binding of AAV1, AAV4, AAV5, and AAV6, while simultaneously enhancing AAV9 binding.^19^ In Alzheimer’s disease, glycoproteomic studies of brain tissue have revealed reductions in galactosylation, fucosylation, bisected, and antennary glycan complexity.^7^ A decrease in galactosylation could significantly diminish terminal galactose species, potentially impairing AAV8 and AAV9 binding. Another limitation of this study is the fact that it was performed using cortical brain organoids and iCardiomyocytes, rather than original tissue samples; determining whether similar glycosylation changes exist in patient tissue samples is crucial to ensure that *in vitro* data align with clinical data. Collectively, this information is essential for identifying specific glycan structures affected in human diseases with aberrant glycosylation, which is crucial for assessing AAV binding capacity and optimizing therapeutic efficacy.

We used CDG as a model to investigate how faulty glycosylation might influence AAV-glycan binding. Up until now, only one AAV-mediated therapy has been tested for CDG: AAV9-PGM1 has been administered intravenously to mice^20^ While AAV transduction efficiency was not assessed, PGM1 protein levels did increase after AAV9 transduction, suggesting that AAV therapy was at least somewhat effective. When AAV9 is administered intravenously, its transduction efficiency in target organs also depends on blood clearance rates (e.g., interactions between AAV proteins and non-target tissues). Consequently, AAV transduction rates in CDG patients might actually increase and not decrease if blood clearance is reduced. However, the majority of AAV subtypes (AAV1, AAV2, AAV6, AAV7, and AAV8) are much less affected by blood clearance compared to AAV9.^21^

Potentially, glycosylation aberrancies leading to impaired AAV binding, might explain why AAV2-neurotropic growth factor (NGF) treatment showed limited efficacy and barely targeted the intended cell type—cholinergic neurons—in individuals with Alzheimer’s disease.^22^^,^^23^ Expanding the evaluation of AAV binding efficacy to a broader range of diseases with secondary glycosylation defects could provide a clearer understanding of the scope of this problem. These insights could also guide strategies to overcome these challenges, such as optimizing AAV dosage or selecting alternative AAV subtypes better suited to the glycosylation profile of the target tissue.

## Materials and methods

### Glycoproteomics and proteomics analysis

Currently, there are no Federal Drug Agency (FDA)-approved therapies for CDG. Over the past decade, patient-derived disease models of CDG patients have been used to study novel treatments, with glycoproteomics as the primary outcome measure. For this study, we included PMM2-CDG, the most common CDG, with neurological symptoms consistently present in all cases.^11^ (Glyco)proteomics data from PMM2-CDG cortical brain organoids were extracted from Radenkovic et al.^24^ ALG13-CDG and PGM1-CDG are other common CDG types, which manifest in one specific tissue type, i.e., the brain (ALG13-CDG) and heart/muscle (PGM1-CDG). As such, we extracted (glyco)proteomics data from these affected tissues from the Proteome Discoverer database (PRIDE: PXD057756) and from Radenkovic et al.^25^

### AAV-binding glycan identification

AAV-binding N-glycan species (≥10% binding efficiency) for AAV1, AAV4, AAV5, and AAV6 were obtained from Mietzsch et al.,^16^ and their structures were manually drawn using the substructure tool from GlyGen. N-glycan species expressing the specific substructure at the terminal end of their glycan species were matched to the glycoproteomics datasets. Plasma membrane localization was determined via the Human Protein Atlas (assessed on 12/16/2024).^26^ AAV-binding glycan enrichment was evaluated using gene set enrichment analysis (GSEA) preranked (MSigDB GSEA v.4.3.3, 100,000 permutations, false discovery rate [FDR] was used for *p* value correction).^27^ AAV cofactor abundance was assessed by filtering the proteomics datasets on the cofactor protein names, and extracting the log2 fold change and unadjusted *p* value. Assessment of proteins that facilitate transduction and transport was performed by extracting the 75 essential genes from Meyer et al.,^6^ and performing GSEA using a preranked list based on log2 fold change values with similar parameters as glycoproteomics.

## Data availability

(Glyco)proteomics data from PMM2-CDG cortical brain organoids were extracted from Radenkovic et al.^24^ (Glyco)proteomics data from PGM1-CDG iCardiomyocytes were exrracted from Radenkovic et al.^25^ Glycoproteomics from ALG13-CDG was extracted from the Proteome Discoverer database (PRIDE: PXD057756).

## Acknowledgments

This study was supported by 1U54NS115198-01 from the National Institute of Neurological Diseases and Stroke (NINDS), the 10.13039/100006108National Center for Advancing Translational Sciences (NCATS), the 10.13039/100009633National Institute of Child Health and Human Development (NICHD), and the Rare Disorders Consortium Disease Network (RDCRN).

## Author contributions

Conceptualization, I.J.J.M. and T.K.; software, I.J.J.M.; formal analysis, I.J.J.M.; investigation, I.J.J.M., R.B., S.R., R.S., and A.P.; writing – original draft, I.J.J.M. and T.K.; writing – review & editing, I.J.J.M., T.K., and E.M.; visualization, I.J.J.M.

## Declaration of interests

The authors declare no competing interests.
